# Plant-Origin Compounds and Materials for Advancing Bone Tissue Engineering and 3D Bioprinting: Traditional Medicine Aspects and Current Perspectives

**DOI:** 10.1155/term/2812191

**Published:** 2025-01-07

**Authors:** Jyrki Heinämäki, Oleh Koshovyi, Iryna Botsula, Alina Shpychak, Hung Quoc Vo, Hoai Thi Nguyen, Ain Raal

**Affiliations:** ^1^Institute of Pharmacy, Faculty of Medicine, University of Tartu, Tartu, Estonia; ^2^Department of Pharmacognosy, National University of Pharmacy, Kharkiv, Ukraine; ^3^Faculty of Pharmacy, University of Medicine and Pharmacy, Hue University, Hue, Vietnam

**Keywords:** bioink, bone 3D bioprinting, bone tissue engineering, medicinal plant, plant-derived biomaterial, plant-origin compound

## Abstract

Bone defects are becoming a true challenge in global health care due to the aging population and higher prevalence of musculoskeletal disorders. The interest in using plant-origin compounds and plant-derived biomaterials in bone tissue engineering (BTE) has been increased due to their availability (abundance), safety, biocompatibility, biodegradability, and low cost. Plant-origin compounds have supportive effects on bone tissue healing, and cell-laden plant-derived biomaterials can be applied in formulating bioinks for three-dimensional (3D) bioprinting to facilitate the preparation of native bone tissue–mimicking structures and customized bone scaffolds. Such plant-derived materials also have the capacity to improve cell viability and support osteoconductive and osteoinductive properties of a bone construct. In this article, we review the ethnomedical aspects related to the use of medicinal plants and plant-origin bioactive compounds in bone healing and the recent developments in the 3D bioprinting of bone constructs with plant-derived biomaterials for advancing BTE. The commonly used 3D-bioprinting techniques, the properties of plant-origin compounds and biomaterials (for bone 3D bioprinting), and the selective examples of bone scaffolds fabricated using plant-derived biomaterials are discussed with a special reference set on applicability, performance, advantages, limitations, and challenges. Plant-origin compounds, biomaterials, and biomimetic 3D-bioprinted constructs could be the basis for a next-generation BTE.

## 1. Introduction

Bone defects are becoming a global public health issue and a burden on health care due to the aging population [1]. In the United States, the annual cost of treating bone defects has been estimated to be $5 billion [2]. The most common causes for such bone defects are trauma, osteodegenerative diseases, congenital abnormalities, and primary tumor resection [3, 4].

Today, the common strategy for treating bone defects is the implantation of bone graft substitutes (scaffolds), such as autologous grafts (autografts), allografts, demineralized bone matrices, or xenografts. The bone graft substitute (scaffold) of choice possesses bone regeneration–enhancing characteristics, such as osteoconductivity, angiogenic capacity, cell viability support, and osteoinductivity. In addition, a bone scaffold should possess biocompatible, biodegradable, and nonimmunogenic properties [5]. Autografts (harvested from the patient) are considered the gold standard in bone replacement and repair, having virtually all the bone regeneration–enhancing characteristics listed above. The limitations of autografts, however, are related to donor site availability, expensive procedures, risk of complications (surgical risks), and perhaps morbidity [3, 4, 6, 7]. Therefore, interest in the development and use of synthetic and plant-derived biomaterial–based bone scaffolds (grafts) as alternatives to autografts is steadily increasing.

The rapid evolution of three-dimensional (3D) (bio)printing technologies and bioinks has opened a new avenue for designing and preparing bone graft substitutes and constructs. Such 3D printing technologies enable the fabrication of next-generation (personalized) bone grafts with a predefined composition, size, shape and internal structure. Today, the three most widely used state-of-the-art 3D bioprinting methods in bone tissue engineering (BTE) are inkjet, microextrusion, and laser-assisted 3D bioprinting, and these key methods are comprehensively reviewed by Ashammakhi et al. [3]. 3D-bioprinted bone graft substitutes and scaffolds can be loaded with cells, growth factors, drugs, additives, biomaterials, and/or nanomaterials to support bone regeneration and healing [2, 8, 9]. There is also increasing interest in finding new materials for bone 3D bioprinting; consequently, the applicability of novel plant-origin materials in bioinks and BTE has been under investigation due to their availability (abundance), safety, biocompatibility, biodegradability, and low cost.

Various plants and plant extracts have been applied since ancient times as a source of medicine in preventing and curing human diseases and health disorders. Medicinal plants and herbs have also been used in traditional medicine to support a natural bone healing process for many centuries [10]. Plants are also rich in bioactive compounds and biomaterials applicable in BTE [11]. The interest in using plant-origin compounds and biomaterials such as bioactive molecules (drugs), phytochemicals, essential oils, biopolymers, polysaccharides, and proteins in BTE has been increased due to their availability (abundance), safety, biocompatibility, biodegradability, and low cost. The advantages of plant-origin bioactive compounds and biomaterials in bone 3D bioprinting and bone tissue regeneration are well documented [5, 12–16]. Many plant-derived polymers and polysaccharides have hydrogel-forming, self-assembly, and crosslinking properties, thus making them promising carrier materials for BTE 3D-bioprinting applications. Moreover, plant-derived biomaterials are capable of supporting bone cell viability and function, which is crucial in bone regeneration and healing [17, 18]. Today, plant-derived biomaterials provide an interesting set of alternative materials for the formulation of bioinks and hydrogels for both cellular and acellular bone 3D bioprinting [11, 12, 16].

The present review article gives an overview of plant-origin compounds and biomaterials applicable in BTE with the main emphasis on their potential uses in bone 3D bioprinting. The main focus of the review article is to gain knowledge of the potential advantages, challenges, and current uses of such compounds and biomaterials in bone 3D bioprinting. The review article also provides historical and ethnomedical perspectives on modern bone 3D bioprinting by presenting the selected examples of the use of medicinal plants and essential oils in bone healing. This review article was compiled using the Google Scholar, Scopus (Elsevier), and PubMed (United States National Library of Medicine [NLM]) databases.

## 2. Bone Structure, Physiology, and Tissue Formation

Bone can be classified as a micro- and nanocomposite system with a unique and highly specialized architecture and composition [19]. Bone tissue is composed of both inorganic minerals (45%–60%) and organic compounds (20%–30%) and additionally a relatively small amount of water (10%–20%) [20]. The calcium phosphate minerals (hydroxyapatite [HA]) as an inorganic part strengthen and support the organic matrix composed of Type I collagen, which in turn is responsible for bone's rigidity, viscoelasticity, and toughness [19–21]. A bone matrix containing noncollagenous proteins (i.e., noncollagenous glycoproteins, bone-specific proteoglycans, and growth factors) has a significant role in affecting bone mineralization and in connecting bone tissue cells and matrix to bone structure–strengthening proteins [20].

Bone is a biologically versatile tissue comprising four cell types: osteoblasts (OBs), osteocytes (OCs), osteoclasts (OCLs), and bone-lining cells. Each of these four cell types has a specific and important role in bone formation and development [22]. OBs are derived from mesenchymal stem cells (MSCs), and their main function is to synthesize bone matrix during bone development and modeling [23]. These cells are responsible for the synthesis of Type 1 collagen, osteocalcin, osteonectin and osteopontin, proteoglycans, and alkaline phosphatase that take part in bone hemostasis and its mineralization and remodeling [19, 20]. OCs are the most abundant bone cells and make up nearly 90%–95% of all bone cells [20]. These cells are responsive to mechanical load and they undergo apoptosis in a bone defect in response to stress. This sensitivity facilitates the involvement of OCLs in the repair of damaged bone. In addition, OCs secrete sclerostin protein, which plays a key role in suppressing OB differentiation and bone formation, ultimately leading to a reduction in bone mass [19]. OCLs originate in monocyte/macrophage lineage precursor cells [23]. They are responsible for bone remodeling by settling in line with the surface of the bone, thus degrading and absorbing old bone matrix. They also produce acids and enzymes, which facilitate the dissolution of both the bone mineral and the organic matrix [19]. According to the literature, the hyperactivity of OCLs is associated with the development of numerous bone diseases, such as rheumatoid arthritis, osteoporosis, osteopetrosis, cancer with metastatic tumors, and alcoholism-derived bone loss [23]. The fourth cell type, bone-lining cells, covers the surface of the bone, and its primary function is to stabilize hematopoietic stem cells and control the influx and efflux of minerals in the regions where the bone comes into contact with other tissues [19, 20].

As a whole, bone remodeling is a highly organized and complex process characterized by the replacement of the old bone with the new one. The process is initiated by the bone resorption carried out by OCLs and followed by the transition period and the final bone formation by OBs [22]. The OBs' orchestrated synthesis of bone matrix is divided into the following main stages: (1) the secretion of collagen proteins, noncollagen proteins, and proteoglycan; (2) the mineralization of the bone matrix, since matrix vesicles bind to organic components and proteoglycans immobilize calcium ions; and (3) the formation of HA crystals by calcium ions and phosphate [22].

Osteogenesis is regulated by several integrated signaling pathways, including transforming growth factor-beta (TGF-β), bone morphogenic protein (BMP), wingless and int-1 (Wnt), hedgehog (HH), notch, parathyroid hormone–related protein (PTHrP), and fibroblast growth factor (FGF) [24]. The Wnt/*β*-catenin pathway [25] plays an important role in promoting OB differentiation and maintaining bone mass; its activation results in the determination of body axis, the induction of the proliferation and differentiation of cells, and morphogenetic signaling [26]. The TGF-β pathway regulates bone cell differentiation and bone remodeling [27]. The use of biomimetic materials that replicate the bone microenvironment can stimulate this signaling pathway, thus improving regeneration. Activation of the BMP signaling pathway simultaneously regulates OB differentiation and results in the transcription of osteocalcin, the collagen alpha-1(I) chain, and alkaline phosphatase [25, 28]. The notch pathway directly inhibits OB differentiation and indirectly influences OCL differentiation and affects the processes of cartilage formation, bone formation, and bone resorption [29].

Plant-origin compounds and biomaterials have been shown to support cellular-level bone repair mechanisms by contributing to bone cell attachment, proliferation, and differentiation in animal studies and consequently enhancing bone remodeling and regeneration [30, 31].

## 3. Traditional Medicine and Ethnomedical Aspects of Medicinal Plants in Bone Healing

### 3.1. Traditional Chinese Medicine (TCM)

TCM has perhaps the longest history of using medicinal plants in bone healing, thus providing long-term evidence on the use, safety, and efficacy of such plants. Therefore, TCM has a long experience in clinical usage for treating bone disease even today [32].

Ancient Chinese scholars observed yin and yang as opposing yet complementary, interdependent and exchangeable aspects of nature. Moreover, it is believed in TCM that everything in nature is affected by the five basic elements (fire, water, wood, metal, and earth) and that the universe is in a constant state of change and, consequently, moving toward dynamic balance or harmony. This knowledge in TCM has been applied to comprehend, prevent, and treat diseases [33]. One of the most well-known and renowned practitioners of TCM is *Hua Tuo (circa* 140–208 A.D.) [34]. He is acclaimed as a divine doctor not only in China but also in culturally connected countries such as Vietnam, Japan, and Korea. Hua Tuo and Li Shizhen (1518–1593), the authors of the “*Compendium of Materia Medica (Bencao Gangmu)*”, are considered two of the most eminent TCM physicians in history.

According to the TCM philosophy, pathological symptoms of bone fracture encompass pain, redness, swelling, and circulatory stagnation, which all delay bone healing. Herbal remedies may be utilized to enhance bone fracture healing by regulating inflammation, promoting blood circulation, and stimulating bone regeneration [35]. Examples of the written prescriptions of Hua Tuo [36] for the treatment of musculoskeletal injuries (translated by Tuong Quan) are presented in more detail in Appendix A.

Scientists have investigated TCM ingredients for their pharmacological effects. Over 30 plant extracts or combinations support bone regeneration and antiosteoporosis [32]. A paste of six herbs promoted bone fracture healing in rabbits by reducing inflammation, improving circulation, and enhancing bone formation [37]. In addition, Zuo Gui Wan, Bushen Tiaogan Formula, resveratrol, *Astragalus membranaceus*, and others help prevent and treat osteoporosis by promoting osteogenic differentiation of aging MSCs. More research, however, is needed on their mechanisms for improving MSC function and preventing aging-related diseases [38].

### 3.2. Traditional Vietnamese Medicine (TVM)

TVM has a rich history (like TCM). TVM incorporates a variety of indigenous folk healing, herbal remedies and practices that have been shaped by influences from other Asian countries such as Japan, India, and especially China [39]. In northern Vietnam, this influence is attributed to factors such as geographical similarity, a shared history of over a thousand years under Chinese dominance, and a language with a common writing system. Conversely, in southern Vietnam, the influence is more attributed to India, which is why the geographical term “Indochina” is used [40]. TVM, however, still possesses distinct features derived from its unique geographical location, climate, and culture. Two prominent medical practitioners who have significantly influenced the development of TVM are Nguyen Ba Tinh, also known as Tue Tinh (1330–1400?), and Le Huu Trac, also known as Hai Thuong Lan Ong (1724–1791) [40–42]. Although they both used the Huangdi Neijing (an ancient collection of Chinese medical texts) in diagnosing illnesses and as the core element in medical theory, they both applied it flexibly in the treatment of Vietnamese individuals [43].

Tue Tinh, renowned for the famous phrase “Southern medicine treats Southern people,” distinctly differentiated between northern Thuoc Bac (TCM) and southern Thuoc Nam (TVM) [40]. He observed two crucial distinctions. Some remedies effective in the North (China) had opposite effects in the South (Vietnam). Tue Tinh recognized Vietnam's tropical climate as distinct from China's temperate climate, thus influencing its plant resources. Considering this, he prescribed minimal doses of “calorific” herbal ingredients [40]. In the case of fevers, he used a “reconciling remedy” (Hoa Giai), initially with soothing agents and later with acrid agents [41].

Like Tue Tinh [40], Lan Ong customized his medical practices for the Vietnamese people. He utilized numerous Vietnamese plants to treat diseases and disorders, thus indicating a departure from TCM heritage in favor of methods better suited to the Vietnamese context [41]. Remarkably, during the UNESCO General Conference's 42^nd^ session in November 2023, a resolution was approved to commemorate the 300th anniversary of Le Huu Trac's birth in 2024, alongside 52 other distinguished personalities and historical events [44].

According to TVM, the basic principles for treating a fractured bone involve three main points: realignment, tight binding, and promoting movement. The first step is to restore the bone to its original position so that the two ends of the bone are aligned. However, the use of surgical procedures in orthopedics to fix the position may damage the periosteum and the vascular system around the fracture site, thus affecting the self-adjusting ability of the fractured bone and hence delaying or nonuniforming bone healing. Therefore, the closed reduction of fractured bones as a prioritized method in traditional medicine has the significant advantage of limiting additional trauma to the fractured bone area, reducing pain for the patient and facilitating prompt bone union and functional recovery [45].

After performing fracture reduction, a common Western approach involves immobilizing the fractured area with casts made of plaster. However, as the plaster dries and swelling reduces, gaps may form, and joint immobilization can contribute to potential deformities at the fracture site. Therefore, TVM believes that immobilizing a fractured position with splints made from materials such as bamboo or wood and cushioning with paper is a more suitable method. The rigid splints and paper cushioning allow the bone's position to be maintained after realignment without displacement. Moreover, the ability to flex and extend muscles is retained, thus creating the pressure that brings the fractured bone ends close together, promoting bone growth and rapid union. External (topical) and oral medications are often combined for several purposes as follows: to control bleeding, alleviate pain, reduce inflammation and swelling, prevent infection, promote bone regeneration, and supplement blood fluids [45].

One of the leading textbooks on TVM, “Traditional Medicine of Vietnam,” lists at least nine species of medicinal herbs applied in the treatment of bone fractures. These plants are *Drynaria fortunei* (*D. roosii* Nakaike), *Psychotria montana* (*Eumachia montana* (Blume) I. M. Turner), *Mussaenda pubescens* Dryand., *Hiptage madablota* (*H. benghalensis* var. *benghalensis*), *Gossampinus malabarica* (*Bombax ceiba* L.), *Calophyllum inophyllum* L., *Argyreia acuta* (*A. obtusifolia* Lour), *Syzygium resinosum,* and *Camellia sasanqua* (*C. oleifera* C. Abel) [46]. The examples of TVM remedies [40, 47–51] intended for the external (topical) or oral treatment of bone fractures are presented in Appendix B. The present examples are (I) documented in ancient medical literature, (II) collected from the experiences of TVM practitioners today, or (III) classified as the other TVM and Asian remedies documented. According to the notes of Tue Tinh in the work “Miraculous Effects of Southern Medicine” (“Nam Dược Thần Hiệu”) [40] and Le Huu Trac in the book “Practice of the Lazy Master of Hai Thuong” (“Hải Thượng Lãn Ông Y tông Tâm lĩnh”) [47], in the case of a fall or injury with a broken bone, the treatment can be arranged as described in Appendix B.

In the TVM, the rhizome of *Drynaria fortunei* (*D. roosii* Nakaike, Polypodiaceae) is commonly used to treat bone fractures and bone pain. In fact, the dried rhizome of *D. fortunei* was named Gu Sui Bu because of its bone-related therapeutic effect. It has a bitter taste and warm property, which is attributed to the heart and kidney meridians. According to ancient pharmacological texts, this herb can detoxify bone-related toxins, alleviate pain from blood stasis caused by “bad qi,” cure the depletion of the five Zang organs (kidneys, lungs, spleen, liver, heart), cure extreme deficiency involving six essential components (vital essence, bones, blood, tendons, semen, and qi), and treat paralysis and numbness in limbs [48]. There are many herbal remedies containing Gu Sui Bu (*Rhizoma drynariae*) as described in Appendix B [48, 49].

In summary, traditional Asian medicine and TVM in particular hold a rich treasure trove of experience in treating musculoskeletal injuries. This serves as a valuable source of inspiration for future research aimed at elucidating its effectiveness in the bone healing process.

### 3.3. Traditional Estonian Medicine (TEM)

Although the history of TEM in Estonia goes back thousands of years, we will discuss the professional use of medicinal plants from the 15th century as the oldest community pharmacies in Estonia were first mentioned in 1422 (Tallinn) and 1430 (Tartu). Most medicines of that time were based not only on medicinal plants but also to a lesser extent on animal products and minerals [52]. In TEM, depending on the health problem, 43%–92% of the remedies used were herbal. In total, around 400 medicinal plants were known in folk medicine, and just about 100 of them were used officially and in pharmacy and medicine in the Soviet era (1940–1991) [53, 54]. Nowadays, one Estonian inhabitant uses an average of 20 cups of herbal tea annually. A total of 88% of respondents used herbal drugs occasionally, and approximately 31% of them even weekly [55].

TEM also describes many applications of medicinal plants in bone healing. The search in the Estonian ethnomedical database Herba [56] provides numerous matches for the keyword “luumurd” (bone fracture), and only two matches which are not related to the direct biological effect of the folk remedy. According to the “magical method of treatment,” the healing of a broken bone was supposed to be facilitated by drawing five pentagonal crosses on a white birch (*Betula* spp.) tree and tying the broken place with it. The bark of the linden (*Tilia* spp.) tree was also suitable for fixing a fractured bone.

It was believed that “coffee from the acorns of the oak (*Quercus robur* L.) is a herbal treatment for bone weakness and anemia” [56]. The term “weakness” refers to a condition in which bones break easily, although the tannins contained in oak bark do not have a direct relationship with bone strength. In conclusion, TEM contains more beliefs and implicit methods than evidence-based therapies in promoting fracture healing. However, we can find them in connection with other medicinal plants known in the Western world.

### 3.4. Other Herbal Traditions and Current Studies

**Figure 1:**
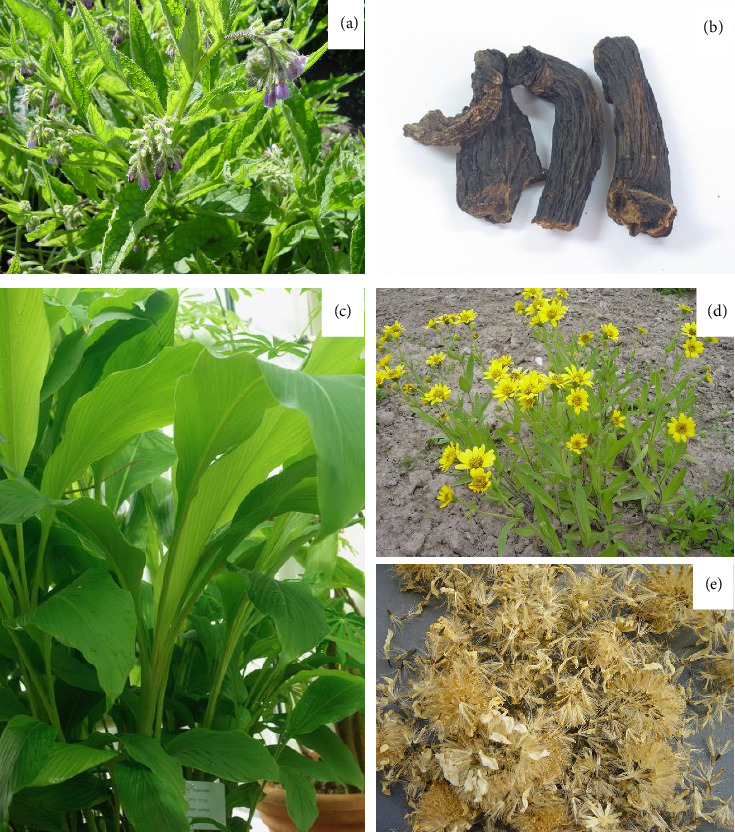
Photographs on (a) common comfrey plant (Symphytum officinale), (b) comfrey roots, (c) Curcuma longa (the ginger family Zingiberaceae), (d) Arnica chamissonis, and (e) Arnica flowers (photos: Ain Raal).

Common comfrey (*Symphytum officinale* L.) (Figure 1) has been known as a medicinal plant to treat bone fractures in traditional medicine in many countries for centuries [57–59]. Its effect was not known in Estonian ethnopharmacology, but it was known as “comfrey tea as a remedy for bone pain” [56]. Comfrey root, aerial parts, and leaves are related to bone tissue as indicated by its ancient name “knitbone” due to the ability to promote callus formation and wound healing. The Latin name of the herbal drug *Consolidae radix* (comfrey root) refers to the same “consolidating” effect [60]. According to the famous English botanist, herbalist, and physician Nicholas Culpeper (1616–1654), outwardly applying comfrey roots is especially good for ruptures and broken bones, and it is said to be powerful in consolidating and knitting tissues together [61].

Dey et al. [58] showed that homoeopathic doses (especially undiluted mother tincture) of comfrey have the potential to enhance osteogenesis in MSCs *in vitro*. The activity of comfrey homoeopathic concentration 6 cH was studied on the removal torque and radiographic bone density around animal titanium implants [62]. After 56 days, the authors concluded that the homeopathic dose of comfrey enhances bone formation around titanium implants at the early stages of osseointegration. Vaezi et al. investigated the ability of the same homoeopathic comfrey dilution (6c) to induce osteogenic differentiation of rat bone-marrow–derived MSCs [63]. The authors found that the preparation is capable of improving osteogenic differentiation and it could have a potential use for the treatment of bone defects.

The osteoregenerative and anti-inflammatory properties of a paste containing comfrey ethanolic extract and calcium hydroxide were studied in treating teeth with chronic granulating apical periodontitis in rats and children [64]. The authors reported that the paste promotes bone regeneration and stimulates osteosynthesis. The allantoin (up to 1.5% in roots) content explains the therapeutic effect of comfrey, which promotes the formation of granulation tissue and callus and accelerates the regeneration of cells due to its effect on promoting cell proliferation [60, 65, 66].

Allantoin in interaction with the alkaloids consolidine and symphytocynoglossin stimulates the growth of fibroblasts, which have a significant role in tissue healing as they contribute to the creation of new connective tissue [67]. On the other hand, the content of hepatotoxic, carcinogenic, and mutagenic pyrrolizidine alkaloids (up to 0.7% in dried roots) limits the internal use of comfrey (*Symphyti radix*, 1999). It is also important to note that comfrey reduces the swollen parts in the immediate neighborhood of fractures [68]. By the German Commission E, the comfrey aerial parts are suggested for the treatment of blunt injuries [69]. In several modern collections of herbal monographs, comfrey is mentioned as a remedy applied topically for ulcers, wounds, and fractures. Due to the content of rosmarinic acid, comfrey also has anti-inflammatory activity, demonstrated *in vivo* [65, 70, 71].

Medicinal plants are rich in potential therapeutic agents applicable for BTE [31]. Curcumin is a dietary polyphenol of the rhizome of ginger (*Curcuma longa* L., *Zingiberaceae*) (Figure 1) with antioxidant, anti-inflammatory, anticancer, and antimicrobial properties. Curcumin has been shown to mitigate bone loss by upregulating bone formation and suppressing oxidative stress [72]. Curcumin also has an impact on neuronal stem cell differentiation and thus regulates various targets, such as nuclear factor kappa-light-chain-enhancer of activated B cells (NF k-β), which is associated with the bone remodeling process [6, 73]. High doses of curcumin can achieve effects equivalent to estrogen in inhibiting bone resorption, promoting bone reconstruction, improving bone mechanical strength, and ultimately preventing postmenopausal osteoporosis [72].

The main component of fresh ginger 6-gingerol showed a joint-protective effect in the model of experimental arthritis by the inhibition of joint inflammation [6]. Funk et al. investigated gingerols and essential oils (GEO) as the secondary metabolite of ginger extracts, and they showed that such plant-origin substances have the ability to block granulomatous inflammation [74]. Furthermore, GEO (28 mg/kg/d intraperitoneal) was shown to prevent a chronic joint inflammation in rats with streptococcal cell wall–induced arthritis, and the effect was at the same level as was obtained with phytoestrogen 17-β estradiol (200 or 600 μg/kg/d subcutaneous).

Blueberries, raspberries, grapes, and peanuts contain a natural polyphenol resveratrol, which is shown to support bone tissue homeostasis, osteogenesis, and bone formation, and has a protecting effect on the potential viability of MSCs [73]. Natural polyphenol resveratrol also has an inhibitory effect on osteoclastogenesis and bone resorption and an antioxidative effect on bone cells [38, 75].

Epigallocatechin-3-gallate (EGCG) is a phenolic substance of green tea, which inhibits bone resorption and stimulates OB activity. Lin et al. investigated the impact of the local use of EGCG on the expression of BMP-2, and they found that such EGCG treatment (in total 0.52 μg/kg/time) accelerated bone matrix formation and provided the strong expression of BMP-2 [76]. BMP-2 is a powerful osteogenic factor that induces OB differentiation and promotes bone formation [76]. German Commission E approved the external use of arnica (*Arnica chamissonis* Less., syn. *A. montana* Hook.) (Figure 1) extracts for injuries and results of accidents, e.g., edema due to fracture [70]. Although Kriplani, Guarve, and Baghael concluded in the review of *A. montana* that it has anti-inflammatory potential, the direct effect of arnica on bone regeneration has not been scientifically proven [77].

## 4. 3D-Bioprinting Techniques and Bioinks

The bone 3D-bioprinting techniques accompanied by computer-aided design (CAD) have taken a giant step within the past 10–20 years. The current printing techniques enable the generation of demanding bone scaffolds in an automated, tailored, and reproducible way. Moreover, next-generation bioactive and biocompatible bioinks have found uses in 3D bioprinting, which enables them to closely mimic the structure of native bone. According to the “diamond concept” of BTE, the bone substitute construct of choice (1) presents a 3D structure with osteoinductive properties, (2) contains osteogenic cells and osteoinductive factors, (3) has sufficient mechanical strength, and (4) is capable of supporting vascularization [19, 78]. Such 3D-bioprinted scaffolds should also facilitate osteointegration, minimize stress shielding, and ensure long-term survival [79].

**Figure 2:**
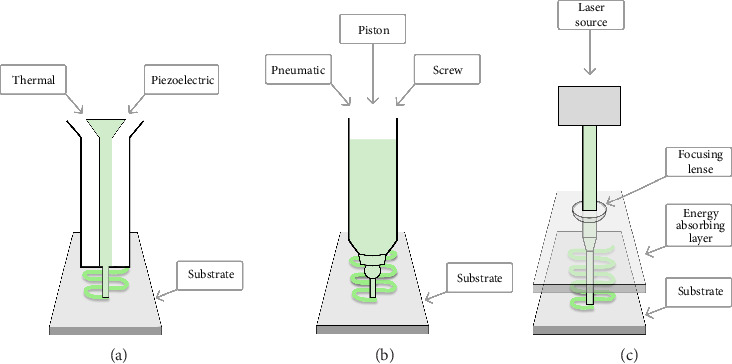
The 3D bioprinting techniques commonly applied in bone tissue engineering (BTE) for generating hydrogel-based constructs: (a) inkjet, (b) microextrusion, and (c) laser bioprinting. Inspired by Ashammakhi et al. [3].

The three 3D-bioprinting methods commonly used in preparing hydrogel-based bone constructs are (1) inkjet, (2) microextrusion (or semisolid-extrusion, SSE), and (3) laser-assisted bioprinting (LAB) (Figure 2). These printing techniques also provide a gelation/crosslinking option, which is crucial for bone 3D bioprinting. According to the literature, microextrusion (or SSE) 3D printing can significantly lower cell viability compared to the other two 3D-bioprinting techniques [3, 80, 81]. Plant-origin hydrogels, however, have been shown to support cell viability by enhancing the 3D microenvironment for the printing process [3, 9]. Moreover, such hydrogels can have a carrier function in the defected area, thus promoting cell proliferation, growth, differentiation, and tissue repair [82].

The formulation of a printable and cytocompatible bioink is crucial for bone tissue 3D bioprinting. Bioinks need to have a viscosity suitable for the 3D printing process and they should be readily crosslinkable to provide structural stability for bone regeneration. The bioink of choice is capable of forming mechanically strong artificial bone structures (extracellular matrix [ECM]) with proper stiffness to enhance cell attachment and proliferation [83]. Bioinks intended for bone 3D bioprinting typically consist of biomaterial (polymer), living cells, growth factors, drugs, bioactive agents, genes, and additives (if needed).

Today, hydrogels are widely used in bone 3D bioprinting since they are readily printable and biocompatible. Hydrogels have also been shown to support cell viability by enhancing the 3D microenvironment for the printing process [3, 9]. Moreover, hydrogels can have a carrier function in the defected area, thus promoting cell proliferation, growth, differentiation, and tissue repair [82]. The most common native-origin hydrogel formers (polymers) applied in bone 3D bioprinting are alginate, gelatin, collagen, and hyaluronic acid. In addition, the use of composite hydrogels and hydrogels based on modified natural polymers (such as gelatin methacryloyl [GelMA]) in bone 3D bioprinting has been described in state-of-the-art literature [9]. As a cellular component, both osteogenic and angiogenic cells have been incorporated into the bioinks for bone 3D bioprinting [3]. For improving osteoinductivity, growth factors such as vascular endothelial growth factor (VEGF) can be used. For supporting osteoconductivity, hydroxyapatite (Hap) and bioactive glass can be added to the bioink. For enhancing mechanical properties, the printed hydrogels can be reinforced with additives, such as thermopolymers, biodegradable ceramics, nanomaterials, and autologous bone particles [3, 9].

## 5. Plant-Origin Compounds and Materials for Advancing Bone Healing and 3D Bioprinting

### 5.1. Plant-Origin Compounds Enhancing Bone Healing

**Figure 3:**
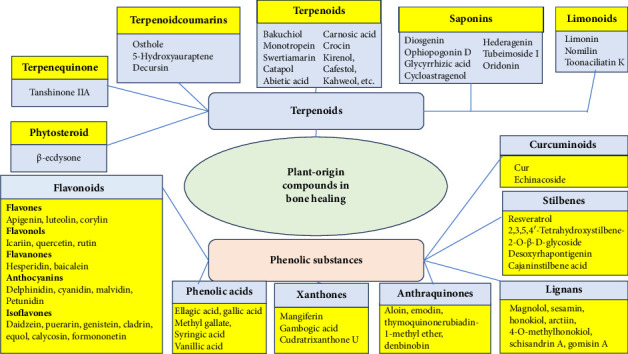
Plant-origin compounds with potential uses in bone healing. The two major groups are terpenoids and phenolic substances.

As mentioned in the previous chapter, traditional medicine holds a rich treasure trove of experience in treating musculoskeletal injuries including bone defects. This serves also as a valuable source of inspiration for finding new plant-origin bioactive compounds for modern bone treatment applications. To date, the bone healing effects of plant-origin compounds have been studied mainly with cell models *in vitro,* and virtually, no preclinical or clinical studies have been performed. Therefore, the use of plant-origin substances in human bone treatment applications can be associated with the risks of allergic reactions and the rejection of such materials. Recently, Okagu et al. [84] published an excellent review article on the molecular mechanisms of action of natural products (including plant-origin bioactive compounds) in preventing and healing bone diseases. With plant-origin compounds, one of the key mechanisms of action in promoting bone healing is through the modulation of bone remodeling signaling pathways and other alternative pathways [84]. Some plant-origin compounds, such as terpenoids and phenolic substances, have found uses in BTE and are able to promote bone healing via several mechanisms.  Figure 3 and the Table A5 in Appendix C show plant-origin compounds with potential effects on bone healing.

#### 5.1.1. Terpenoids

The antiosteoporotic effects of plant-origin terpenoids have been widely reported in the literature highlighting their potential pharmacological uses and molecular mechanisms in osteoporosis prevention and treatment [85]. Many studies have reported that swertiamarin (from *Schultesia lisianthoides*, *Gentiana thunbergii*, etc.), catapol (from *Veronica kellereri*, *Scutellaria racemosa*, etc.), and monotropein (from *Galium rivale*, *Pyrola japonica*, etc.) increase both OB and OC differentiation *in vitro* [86–88]. The treatment of bone disorder with swertiamarin reduced the expression of the receptor activator of nuclear factor kappa-Β (NF-*κ*B) ligand (RANK and RANKL) and significantly increased the expression of osteoprotegerin (OPG) levels, thus leading to an enhanced antiosteoclastogenic effect [87].

The plant-origin terpenoids affecting bone metabolism and reducing bone loss are listed in Appendix C. All of these substances can readily be isolated from different plant raw materials. The bone healing effects of the abovementioned terpenoids were studied with different cell models at several different doses and their pathways and effects were determined [85]. Terpenoids have been reported to show protective activity against bone loss via improving OCL and OB differentiation, but no clinical studies have been performed to date, although the terpenoids' epigenetic role has been demonstrated [87]. Some studies, however, were conducted to provide evidence of the bone healing mechanisms of isolated terpenoids [89].

Several phytochemicals have been shown to control bone homeostasis via a number of transduction pathways on OCLs and OBs influencing bone tissue invert. Plant-origin terpenoids have been shown to influence osteoblastogenesis, although a terpenoid-induced OB differentiation has been not reported [90]. Thus, terpenoid–coumarins affect an upregulated BMP-2 expression, and a few terpenes are capable of stimulating the Wnt signaling pathway. Such signaling pathway controls e.g., cell polarity, cell fate determination, and cell migration. Terpene–quinones and limonoids are capable of stimulating the mitogen-activated protein kinase (MAPK) pathway leading to runt-related transcription factor-2 (RUNX-2) and osterix (OSX) transcription factor overexpression [91]. Ras–MAPK signaling affects osteogenic differentiation and bone formation. The RUNX-2 and OSX transcription factors serve as primary transcription factors in bone formation. The antioxidative activity of terpenoids through the prosurvival transcription factor activation provides OB proliferation and survival, thus enhancing bone deposition. The action of a few terpenes and phytosteroids mimics the action of SERMs, thus revealing estrogen activity in some cells and promoting OB differentiation. Moreover, terpenes and terpene–quinones have the ability to increase OB activity by inhibiting the NF-*κ*B pathway indirectly [85]. The NF-*κ*B protein complex is a transcription factor that quenches bone resorption and accelerates bone formation.

Like polyphenols, all terpenoids have a high antioxidative activity and they have the ability to positively regulate OB differentiation [89, 92]. It has been shown that terpenoids promote SIRT-1 expression, which has a positive impact on the viability, proliferation, and differentiation of OBs and a negative impact on the survival, differentiation, and activity of OCLs [93, 94].

#### 5.1.2. Phenolic Substances

Plant-origin phenolic substances with antioxidative activity (mainly flavonoids) have been shown to have the ability to control bone cells; consequently, such compounds have advantages over traditional therapeutic agents in long-term use [93, 95, 96]. The plant-origin flavonoids with a positive impact on bone metabolism and bone loss are summarized in Appendix C. These flavonoids have been shown to induce OB differentiation and reduce OVX-, LPS-, and HFD-related bone loss. They are also capable of regulating bone homeostasis through the mechanism, where the flavonoids orchestrate the interaction with the signal transduction pathways of bone tissue cells. In addition, flavonoids most likely support osteoblastogenesis, increase OB activity via enhanced bone deposition, and have an inhibiting effect on osteoclastogenesis and OCL activity. According to Sõukand [56], flavonoids possess a prominent antioxidative activity, thus resulting in an increase in OB as well as a decrease in OCL differentiation. These effects are obtained based on epigenetic regulation, such as DNA methylation, miRNA expression, and histone (de)acetylation. Furthermore, SIRT-1 (an important deacetylase) is tuned on by the NAD+/NADH ratio, and phytochemicals with an antioxidative activity promote the activation of SIRT-1. This activation also promotes the viability, proliferation, and differentiation of OBs; induces OCL apoptosis; and inhibits the activity and differentiation of OCLs [93].

The examples of phenolics supporting bone metabolism and reducing bone loss are listed in Appendix C. All of these phenolics control bone homeostasis since they are capable of inducing the interaction(s) of bone tissue cells with signal transduction pathways. They have a significant antioxidant effect and thus enhance the differentiation and activity of OBs and suppress the corresponding activities of OCLs via epigenetic regulation. These substances regulate the increase in reduction potential, thus changing the NAD+:NADH ratio and, as a result, the SIRT-1 activation. SIRT-1 is a major histone deacetylase involved in the heterochromatinization of specific genes in OCLs and OBs, thus leading to a decrease in bone resorption and an increase in bone deposition [94, 97].

### 5.2. Plant-Derived Materials Advancing 3D Bioprinting and Performance of Bone Scaffolds

The biomaterials applied in 3D bioprinting are derived from chemical synthesis or of natural origin. Synthetic polymers provide good mechanical properties and biocompatibility, enable reproducible 3D bioprinting, have controlled degradation, and will not have issues related to acute toxicity or hemolysis [8, 21]. The major limitation of synthetic polymers, however, is the formation of potentially harmful degraded products [20]. The limitations related to the use of synthetic polymers could be overcome by replacing them with plant-derived biomaterials [81, 83, 98–100]. Plant-derived biomaterials reduce the possibility of immunogenicity and exclude ethical issues, which could limit the use of biomaterials of animal or human origin [101]. Plant-derived polymers and polysaccharides are biocompatible and nontoxic materials that have good cellular adhesion and enzymatic degradation ability. Plant-origin proteins possess the ability to manage biological cell attachment, growth, proliferation, and differentiation. Polysaccharides need to be modified to enhance such properties [21]. Natural biopolymers have free degradation rates but their usage can be limited due to the deficiency of mechanical properties of the scaffold such as surface roughness, hydrophilicity, functional group, and surface architecture [5]. Native starches have also shown some limitations mainly due to their hydrophilicity, poor rheology, high swelling, and limited mechanical properties.

#### 5.2.1. Alginate

Alginate is one of the most widely used biomaterials in BTE due to its biocompatibility, low toxicity, easy gelation, and multiple modifications [81]. Alginate is applicable for preparing both solid bone scaffolds and hydrogels. In order to increase the mechanical strength and solidity of a scaffold, alginate is combined with other materials or nanoparticles. Alginates have been used in BTE with polyvinyl alcohol (PVA), HA, calcium silicate, sodium alginate, gelatin, silk fibroin, and graphene oxide. Alginate as a hydrogel former is more suitable for osteogenic differentiation than hyaluronic acid [99].

Bendtsen, Quinell, and Wei used SSE 3D bioprinting for preparing novel alginate–PVA–HA hydrogel-based bone scaffolds of high shape fidelity [102]. The rheological properties and SSE 3D printability of such alginate-based hydrogel formulations were very good. In addition, the novel hydrogel 3D printing formulation developed enabled cell encapsulation and bioprinting of scaffolds with uniform cell distribution (mouse calvaria 3T3-E1 cells). The 3D-bioprinted alginate–PVA–HA scaffolds remained intact for 14 days. The authors concluded that the present osteoconductive and biodegradable alginate–PVA–HA hydrogel formulation is readily SSE 3D bioprintable and the corresponding scaffolds are promising candidates for treating personalized bone defects [102].

The combinations of alginate/gelatin have been shown to enhance the metabolic activity of bone cells and the mechanical properties of BTE scaffolds, and thus to facilitate bone 3D bioprinting [103]. Park et al. [104] investigated *in vivo* bone regeneration after subcutaneous implanting alginate/gelatin/collagen-based 3D-bioprinted scaffolds in mice. The authors reported faster bone regeneration in prevascularized structures (cell-laden hydrogels containing both BMP-2 and VEGF) than in nonvascularized structures (cell-laden hydrogel with no growth factor and cell-laden hydrogel with BMP-2 only). Composite alginate-based hydrogels are also applicable in BTE as a scaffold material. For example, the RGD-grafted composite hydrogel of alginate and chitosan accelerated the bone tissue formation and vascularization [81]. The strengthening of alginate with graphene oxide and gelatin had a positive effect on the compressive strength and swelling rate of a BTE scaffold [5].

Iglesias-Mejuto and García-González (2021) introduced alginate–HA aerogel scaffolds for bone regeneration by combining the SSE 3D bioprinting (with CAD) of the corresponding hydrogels and the supercritical CO_2_ drying of the gels [105]. The alginate–HA aerogel scaffolds prepared were highly porous and biocompatible and supported the attachment and proliferation of MSCs. Moreover, the bone aerogel scaffolds developed enhanced the fibroblast migration toward the damaged bone area, thus supporting bone regeneration. The present alginate–HA aerogel scaffolds could find use as a promising alternative in individualized BTE [105].

The alginate/chitosan-templated HA scaffolds were prepared using a layer-by-layer technique and the final scaffolds presented a high porosity (approximately 77.5%), compression module (1.7 MPa), and relevant degradation rate [5]. It was verified that both human fibroblasts and chondrocytes readily adhered *in vitro* on the surface of such scaffolds and progressed well on the scaffolds. The preparation of such composite scaffolds is also versatile and enables tailoring options. Consequently, alginate combined with the abovementioned materials proved to be the composite material of choice for regenerative TE applications [6]. Recently, the following novel alginate composite scaffolds have been introduced and shown to be promising for BTE applications: alginate/TEMPO-oxidized cellulose 3D scaffolds, oxidized alginate/gelatin polypyrrole: PSS 3D-printed hydrogel, gelatin/alginate/graphene oxide composite scaffolds, TiO_2_-chitosan/sodium alginate scaffolds, and alginate/nano-HA scaffolds [5, 106, 107].

#### 5.2.2. Cellulose and Cellulose Derivatives

Cellulose is the most abundant natural polymer widely used in both pharmaceutics and TE. Cellulose is a linear polymer comprising β-D-glucose. The cellulose ethers, such as methyl cellulose, hydroxypropyl cellulose, hydroxypropyl methylcellulose (HPMC), and ethyl cellulose are well-established and safe excipients in pharmaceutics. Since native cellulose and its derivatives have the ability to create biocompatible constructs (scaffolds) with a high mechanical strength, this makes them interesting plant-origin alternatives for BTE applications [5]. The more recently introduced nanocrystalline, nanofibrillated, and microfibrillated celluloses have also found uses in TE applications [106].

Microextrusion or SSE 3D bioprinting is widely used for cellulose derivatives and nanocellulose since these materials have a number of advantages in formulating the bioinks, especially for bone extrusion–based 3D bioprinting. Methylcellulose (MC) is an inert and temperature-sensitive biopolymer used in preparing porous composite TE scaffolds. MC has been combined with alginate and calcium phosphate cement to increase the viscosity of bioink in bone 3D bioprinting [108]. Carboxymethyl cellulose (CMC) is another cellulose ether used in the BTE applications. The carboxyl group of CMC can become a nucleation site for calcium ions to have a positive impact on bone mineralization. Since CMC is a negatively charged biopolymer, it enables the formation of strong electrical interactions with positively charged polymers (such as gelatin and chitosan), thus enhancing the reliability of the scaffold in BTE applications [106]. CMC has been combined with gelatin and beta-tricalcium phosphate to increase the viscosity of bioink and ensure the macroporosity of the scaffold in bone 3D bioprinting [109]. In addition to MC and CMC, HPMC combined with calcium magnesium phosphate [110] and hydroxyl ethylcellulose (HEC) combined with glycerophosphate, chitosan, and cellulose nanocrystals [111] have been successfully used in formulating bioinks for bone 3D bioprinting.

Cellulose and its derivatives are also well applicable for strengthening natural and synthetic polymers in preparing 3D-bioprinted bone scaffolds. Cellulose derivatives can be combined, for example, with PVA, polylactic acid (PLA), chitosan, and silk fibroin [5]. Bacterial cellulose (BC) has been used in BTE either alone or in combination with collagen to enhance the mechanical properties of bone scaffolds [81]. He et al. used microfibrillated cellulose combined with collagen and HA (Col–HA) in preparing BTE scaffolds [86].

Currently, the application of nanocelluloses is of great interest in BTE. Nanocelluloses can be used as a principal biopolymer in preparing BTE scaffolds, or as a substitute agent in bioinks for the other natural polymer, such as alginate and gelatin. Nanoscale cellulose derivatives are divided into three groups: nanocrystalline cellulose (NCC), nanofibrillated cellulose (NFC), and bacterial nanocellulose (BNC). They differ from each other in terms of particle size, crystallinity, mechanical strength, and in terms of some characteristics related to their function [106]. In general, the structure of nanofibrous composites resembles very much to the structure of natural ECM and especially to the protein fibers of ECM (elastin and fibrillar collagen) [112].

NCC is prepared by washing and milling lignocellulosic biomass, then purifying the biomass via alkali or acid-chlorite treatment, and finally conducting acid hydrolysis [106]. Since NCC contains only crystalline regions, it provides enhanced tensile strength for BTE scaffolds and it is the biomaterial of choice for improving the mechanical properties of scaffolds. Moreover, NCC has numerous biorelevant properties, including biodegradability, biocompatibility, sustainability, consistent rod-like-shaped crystals, large surface area, liquid crystalline behavior, tailored surface chemistry, and safe carbohydrate-based origin [82]. NCC is also capable of strengthening polymeric materials and increasing the mechanical strength of BTE scaffolds. Moreover, NCC can play an important role as a matrix-forming nanomaterial for enhancing OB activity in BTE [100].

To date, however, only a few studies have been published on the use of NCC in bone 3D bioprinting. Maturavongsadit et al. introduced a cell-laden NCC/chitosan/HEC-based bioink for bone SSE 3D bioprinting. The incorporation of NCC enhanced the gelation and viscosity of the composite hydrogel, supported osteogenic cell differentiation, and improved the mechanical properties of the bone scaffold [110]. Nanocellulose blends (NCBs) loaded with NCC were also introduced as hydrogel bioink formers for the SSE 3D bioprinting in bone and cartilage TE [113]. The authors concluded that the rheological, swelling, and biocompatibility properties of such NCB-alginate–based bioinks are favorable for SSE 3D bioprinting.

In recent years, an increasing number of NFC-based bioinks intended for bone 3D bioprinting have been reported in state-of-the-art literature. NFC is prepared by degrading lignocellulosic biomass and it has good biocompatibility, biodegradability, and water retention properties [106]. The mixtures of NFC and alginate in 3D printing bioinks have been shown to be more applicable in cartilage TE than BTE. Im et al. developed a novel osteogenic bioink composed of NFC, alginate, and polydopamine nanoparticles for SSE 3D bioprinting and BTE [114]. The rheological properties (i.e., high viscosity and shear thinning) of such bioink were advantageous for SSE 3D bioprinting. The bone scaffolds generated with the present NFC bioink had an enhanced mechanical strength sufficient for BTE applications [114].

The use of the third nanocellulose derivative (BNC) in bone 3D bioprinting is limited due to the complex protofibrous structure of the material, thus increasing the risk of nozzle clog in 3D bioprinters and leading to negative effects on cell migration [106]. On the other hand, BNC contains the largest amount of water and has the highest water absorption capacity for the survival of cells. In addition, BNC provides a porous nanofiber structure, thus enhancing significantly cell adhesion.

#### 5.2.3. Hyaluronic Acid

Hyaluronic acid is a native-origin unbranched glycosaminoglycan polymer and an integral component of an ECM, thus supporting cell proliferation and migration. Hyaluronic acid is a safe, biodegradable, biocompatible, and nonimmunogenic hydrophilic polymer with the ability to form hydrogels. Therefore, hyaluronic acid has numerous medical applications including (but not limited to) its uses as a soft tissue filler and skin treatment agent, as a biomaterial for curing knee osteoarthritis, and as an active agent for the treatment of dry eye.

According to Ding et al. hyaluronic acid–based hydrogels have found widespread uses as bioinks in the 3D bioprinting of bone, cartilage, nerve tissue, and skin [115]. Hyaluronic acid hydrogels have excellent rheological properties for extrusion bone 3D bioprinting; for example, when combining hyaluronic acid with other biopolymers (i.e., alginate and chitosan), the viscoelasticity of hydrogels can be modified. Crosslinking improves the mechanical properties of hyaluronic acid–based hydrogels in bone 3D bioprinting. The 3D-bioprinted bone scaffolds prepared from hyaluronic acid hydrogels presented good primary cell survival, high angiogenic activity, and excellent spontaneous osteogenic differentiation *in vitro* [115]. Janarthanan et al. introduced a novel hydrogel for bone 3D bioprinting by combining three biocompatible biomaterials, such as hyaluronic acid, hydroxyethyl acrylate (HEA), and GelMA in the bioink [116]. The present bioprinting gel showed stable rheology properties, good printability (in both injection- and extrusion-based 3D printing), biocompatibility, and bone cell viability–supporting properties.

#### 5.2.4. Pectin

Pectin is a well-known plant-origin material composed of the chains of galacturonic acid units linked as 1,4-*α*-glucosides [117]. Pectin is widely used in pharmaceutics and food technology since it is considered a safe, biocompatible, and nonirritating material. High-molecular-weight pectin has been used in BTE as a hydrogel-forming material in composite scaffolds. Pectin is able to form a gel by interacting with divalent cations [31]. Amirian et al. created a composite BTE scaffold based on gelatin–pectin–biphasic calcium phosphate, which was loaded with growth factors, BMP-2 and VEGF [118]. The authors showed with the present BTE scaffold an enhanced preosteoblast spreading behavior and cell proliferation *in vivo* in comparison to the corresponding scaffolds without growth factors. The calcium phosphate ions mimic human plasma for apatite formation [31]. Even though pectin could be a valid biomaterial for bone 3D bioprinting, the research work in this area is still very limited. This could be due to the poor rheological properties of pectin-based bioinks and the limited printability and mechanical properties of the scaffolds [119].

#### 5.2.5. Starch and Starch Derivatives

Starches are extracted from many abundant plants, e.g., from corn (*Zea mays*), wheat (*Triticum aestivum*), rice (*Oryza sativa*), potato (*Solanum tuberosum*), tapioca (*Manihot utilissima* Pohl), and peas (*Pisi amylum*) [117]. Depending on the source of isolation (the plant), vegetable starch contains linear amylose and branched amylopectin at different ratios. The ratio of these two polysaccharides [based on *α*-(D)-glucose] determines the crystallinity of starch (amylose showing a high tendency for crystallization) [117].

Despite being a potential biomaterial for BTE, starch has some limitations in bone repair owing to its fragility, low surface area, and sensitivity to water. In order to tackle such drawbacks, natural and synthetic polymers, such as ceramics, were added to starch-based bone constructs [82]. Guo et al. showed that the incorporation of HA or vinyl monomers enhanced the mechanical strength of starch-based bone scaffolds [82]. The combination of starch with PCL accelerated the bone formation and enhanced the bone repair. The composite BTE scaffold of starch and chitosan was found to promote the ALP activity of the OB-like cells [82]. Moreover, as the composite starch-PCL scaffolds were modified with silanol groups, the present treatment fostered the osteogenic differentiation of stem cells [120]. The ternary mixture of starch, chitosan, and HA increased the mechanical strength of the scaffold, thus making such composite scaffold more suitable for hard tissue engineering [82].

Native starches have also shown some limitations in bone 3D bioprinting mainly due to their hydrophilicity, poor rheology, high swelling, and limited mechanical properties. To overcome these deficiencies, Koski and Bose (2019) developed bone 3D-printed starch–HA composite scaffolds and investigated the effects of the amylose content of starch on the mechanical and physical properties of scaffolds. Native corn, potato, and cassava starches were used as plant-origin material components in the bioinks. Amylose was shown to be a biologically active reinforcement moiety of such 3D-printed composite bone scaffolds, thus enhancing the mechanical properties of the scaffolds [121]. More recently, Invernizzi Sponchiado et al. modified potato starch for use in the modular extrusion 3D printing of bone bio-scaffolds. Dry heating treatment (DHT) was used to modify native potato starch. The use of DHT-modified potato starch improved the 3D printing performance of the hydrogel bioink and the 3D-printed bone scaffolds presented good mechanical properties, limited biodegradability, and low swelling [122].

#### 5.2.6. Carrageenan

Recently, Chimene et al. [123] developed an osteoinductive nanoengineered ionic-covalent entanglement (NICE) bioink for 3D bone bioprinting. The NICE bioink is composed of covalently crosslinkable GelMA, ionically crosslinkable kappa-carrageenan, and electrostatically charged nanosilicates. The combination of crosslinked GelMA and kappa-carrageenan results in the formation of an ionic-covalent entanglement network. The role of carrageenan was also to increase the viscosity of the bioink and prevent flow as the bioink cools. The NICE bioinks enabled high printing performance, good mechanical properties, and enzymatic degradation characteristics. The next-generation NICE bioink was successfully used for preparing patient-specific, implantable 3D scaffolds for the repair of craniomaxillofacial bone defects [123].

#### 5.2.7. Polyhydroxyalkanoates (PHAs)

PHAs are nontoxic biocompatible, biodegradable native-origin biopolyesters. PHAs are capable of forming films and have adjustable mechanical properties. The films composed of the fifth generation of such biopolyesters (3-hydroxybutyric acid-*co*-3-hydroxyvaleric acid-co-3-hydroxyhexanoic acid trimer, PHBVHHx or PHBVHx) were reported to promote the growth of human bone marrow MSCs and their differentiation into bone [8].

## 6. Future Directions

The plant-origin bioactive compounds and biomaterials used in bone 3D bioprinting hold great promises due to their unique characteristics such as availability (abundance), safety, biocompatibility, gelation capacity, biodegradability, and low cost. Future research work, however, is needed to gain knowledge of the molecular mechanisms of action of such plant-origin bioactive compounds in supporting bone regeneration. Plant-based bioinks are attractive alternatives for conventional synthetic material–based bioinks, and their advantages are well documented in numerous studies *in vitro*. In spite of an extensive research work, the development of plant-based bioinks for bone 3D bioprinting is still in its infancy, and there are numerous challenges to overcome. The major “bottlenecks” hampering true clinical applications include (but are not limited to) the insufficient biomimicry and mechanical properties, optimization of the 3D bioprinting formulations (i.e., rheology, printability, and cell viability), and the incomplete verification of the performance of the present bioinks and scaffolds *in vivo*. To date, only a few studies have been reported on the validation of such plant-based bioinks *in vivo* and on the scalability for clinical applications. In the future, well-designed *in vivo* studies in animal models and human trials are crucial to verify the performance, efficacy, and safety of plant-based bioinks and 3D-bioprinted tissue engineering scaffolds. As the 3D bioprinting techniques are rapidly developing, it is obvious that this will lead to significant improvements in scaling up and cost-effective manufacturing of plant-based 3D-bioprinted scaffolds. The use of plant-derived nanomaterials-based bioinks for preparing hybrid nanomaterial scaffolds and utilizing decellularized plants for preparing prevascularized scaffolds are the two interesting future directions in plant-based tissue engineering, which could also find uses in bone 3D bioprinting.

## 7. Conclusions

Medicinal plants and herbs are rich in bioactive compounds and materials with potential therapeutic and technological applications in BTE. In traditional medicine, the use of plant-origin compounds and plant tissues to support bone healing has been known for thousands of years. 3D bioprinting has opened a new avenue for the design and fabrication of next-generation bone scaffolds. To date, however, only little is known about the potential and advantages of plant-derived biomaterials in bone 3D bioprinting. Plant-derived biomaterials could be used as alternative materials in preparing biocompatible bioinks for bone 3D bioprinting. Plant-derived biomaterials can be readily combined with synthetic and semisynthetic polymers in such bioinks. Plant-based 3D-bioprinted bone scaffolds are biocompatible, cost-effective, and easy to handle, and they do not have immunogenicity limitations and ethical concerns commonly related to the bone scaffolds of human and animal origin. Plant-origin compounds and biomaterials, however, are often sensitive to higher temperatures and organic solvents and/or water, which causes challenges for bone 3D bioprinting. The next steps in research and development are to find the most feasible formulations for plant-derived biomaterials for bone 3D bioprinting and to investigate the 3D printing behavior and stability of such materials. To date, the bone healing effects of plant-origin compounds and biomaterials have been studied mainly with cell models *in vitro*, and virtually, no preclinical or clinical studies have been performed or published so far. Therefore, the use of plant-origin substances and biomaterials in human BTE can be associated with the risks of allergic reactions, the survival of cells, the rejection of such materials, and the physicochemical interactions with the polymer base and other components. Further studies with animal model(s) *in vivo* are needed to verify the efficacy and safety of plant-origin compounds and biomaterials in BTE.

## Figures and Tables

**Table 1:** The written prescriptions of Hua Tuo (translated by Tuong Quan) [36] for the treatment of conditions related to musculoskeletal injuries.

No	Remedy	Preparation method/administration	Indication	Ref.
1	Fresh leaves of Da Ma Gen (*Cannabis sativa* L.)	Extract the crushed fresh leaves. Drink a small bowl of the obtained liquid. If fresh leaves are not available, dried ones can be used to make a decoction - Use orally	Bone fractures	[36]

2	1. Huang Gou Tou Gu (canine skull of dog)	Use a set of Huang Gou Tou Gu, roast until crisp, then grind into a powder. Grind the dried Mu li. Mix the Huang Gou Tou Gu powder, Mu li powder, and Guan gui powder at a ratio of 5:3:2. Cook rice with water to obtain a porridge broth. Take a clean silk cloth, spread the porridge broth on it, and then sprinkle the powder mixture prepared as above. Wrap it around the broken bone - Use topically	Bone fractures	[36]
	2. Mu li (*concha ostreae*)			
	3. Guan gui (*cortex Cinnamomi*)			

3	Sheng di huang (*radix Rehmanniae*)	Crush Sheng di huang and apply it to the injured area Use topically	Injured tendons	[36]

4	1. Sheng di huang (*radix Rehmanniae*)	Mix 2 g each of finely ground ingredients. Take one spoonful with alcohol three times a day - Use orally	Injured tendons	[36]
	2. Dang gui (*radix Angelicae sinensis*)			
	3. Du Huo (*radix Angelicae pubescentis*)			
	4. Ku Shen (*radix Sophorae flavescentis*)			
	5. Alcohol			

5	1. Meng Chong (*Tabanus bivittatus*, excluding legs and wings)	Use equal amounts of Meng Chong and Mu dan pi (*Cortex Moutan*), then finely grind. Take one spoonful with alcohol each time	Broken wrist and blood stasis	[36]
	2. Mu dan pi C*cortex Moutan*)	- Use orally		

6	1. Da huang (*radix et rhizoma Rhei*) 6 g	Pour 6 L of boiling water, decoct until it reduces to 3 L, divide into 3 doses and take with alcohol - Use orally	Broken wrist and blood stasis	[36]
	2. Gui Xin (*cortex Cinnamomi*) 2 g			
	3. Tao Ren (*Semen Persicae*, peeled) 60 kernels			
	4. Alcohol			

7	1. Tian luo (*Pila polita*)	Crush Tian luo, mix with distiller's grains, and apply thickly around the affected area (leaving a small hole)	Bone fractures with pus	[36]
	2. Distiller's grains	- Use orally		

**Table 2:** Examples of traditional Vietnamese medicine (TVM) remedies intended for the external (topical) or oral treatment of bone fractures. The present examples are documented in an ancient medical literature.

No	Remedy	Preparation method/administration	Indication	Ref.
1	1. Peel of ripe red oranges	Use the oily pith of the peel of ripe red oranges and soak it in concentrated alcohol	Bone fractures	[46, 47]
	2. Alcohol	- Use orally and topically		

2	*Bulbus Allii cepae* (the bulbs of *Allium* cepa var. a*ggregatum,* including roots)	Crush and stir fry bulbs (including the roots) and apply them to the fractured area. Replace with a fresh application when it cools - Use topically	Fracture of limb bones	[46, 47]

3	1. Zao jiao (*fructus Gleditsiae sinensis*)	Mix each ingredient in equal amounts and grind into powder. Squeeze ginger to extract juice, mix it with the powder, and apply to the fractured area - Use topically	Fracture of limb bones	[46, 47]
	2. Ce bai ye (*cacumen Platycladi*)			
	3. Gu Sui Bu (*Rhizoma drynariae*, removed scales)			
	4. Fresh ginger			

4	1. Mu Mian Pi (*Cortex Bombax ceibae*)	Crush Mu Mian Pi, mix with egg whites, add vinegar, and cook it. Add smashed ginger and apply to the dislocated or misaligned bone. Replace the poultice when it has dried until completely healed - Use topically	Bone dislocation or misalignment	[46, 47]
	2. Egg whites			
	3. Vinegar			
	4. Ginger			

5	1. Crushed onions (*Bulbus Allii cepae*)	Crushed onions (mixed with honey, evenly apply, and leave on)	Bone dislocation or misalignment	[46, 47]
	2. Honey	- Use topically		

6	Niu xi (*radix Achyranthis bidentatae*)	Crush Niu xi and apply on the injured area - Use topically	Bone dislocation or misalignment	[46, 47]

7	1. Dan zhu ye (*herba Lophatheri*)	Crush Dan zhu ye with salt, then mix with clear vinegar, fry it, wrap in banana leaves, and apply to the swollen and painful area - Use topically	Bone dislocation or misalignment	[46, 47]
	2. Salt			
	3. Vinegar			
	4. Banana leaves			

**Table 3:** Examples of traditional Vietnamese medicine (TVM) remedies intended for the external (topical) or oral treatment of bone fractures. The present examples are collected from the experiences of TVM practitioners today.

No	Remedy	Preparation method/administration	Indication	Ref.
1	Gu Zhi wan: 1. An Xi Xiang (*Benzoinum*) (80 g)	Grind the mixture into a powder. Set aside An Xi xiang separately and mix it with honey, then add an xi xiang back into the mixture to form pills. Take 10–12 g daily with hot water diluted in alcohol - Use orally	Lower back pain, cramps, and arthralgia	[48, 49]
	2. Niu Xi (*radix Achyranthis bidentatae*) (80 g)			
	3. Gu Sui Bu (*Rhizoma drynariae*) (40 g)			
	4. Bu Gu Zhi (*fructus Psoraleae*) (80 g)			
	5. Bing Lang (*semen Arecae*) (80 g)			
	6. Gui Xin (*cortex Cinnamomi cortex*) (80 g)			

2	Bu Jin tang: 1. Bai Shao Yao (*radix Paeoniae alba*) (10 g)	Decoct and take one dose per day, divided into two servings - Use orally	Sprains and dislocations	[48, 49]
	2. Mo yao (*Myrrha*) (6 g)			
	3. Hong hua (*flos Carthami*) (8 g)			
	4. Ding xiang (*flos Caryophylli*) (6 g)			
	5. Chen pi (*pericarpium Citri reticulatae*) (8 g)			
	6. Gu Sui Bu (*Rhizoma drynariae*) (12 g)			
	7. Fu ling (*Poria cocos wolf*) (12 g)			
	8. Shu di huang (*radix Rehmanniae preparata*) (10 g)			
	9. Ru xiang (*gummi Olibanum*) (6 g)			
	10. Dang gui (*radix Angelicae sinensis*) (12 g)			

3	Gu She Nei Fu fang: 1. Gu Sui Bu (*Rhizoma drynariae*) (12 g)	Decoct and take one dose per day, divided into two servings, mixed with alcohol - Use orally	Promote blood circulation and bone marrow regeneration	[48, 49]
	2. Wu jia pi (*cortex Acanthopanacis*) (10 g)			
	3. Ru xiang (*gummi Olibanum*) (8 g)			
	4. Zi ran tong (*Cuprum*) (10 g)			
	5. Xue jie (*sanguis Draconis*) (8 g)			
	6. Xu duan (*radix Dipsaci*) (12 g)			
	7. San qi *(radix et rhizoma Notoginseng*) (8 g)			
	8. Mo yao (*Myrrha*) (6 g)			
	9. Dang gui (*radix Angelicae sinensis*) (12 g)			
	10. Hai tong pi (*cortex Erythrinae*) (10 g)			

4	Zou Ma san: 1. Gu sSui Bu (*Rhizoma drynariae*)	Use 12 g of each ingredient, crush the herbs into a fine powder, and use twice a day. It can be consumed by brewing with boiling water or applied externally after grinding into a paste - Use orally or topically	Soft tissue injuries and closed fractures	[51]
	2. Fresh He ye (*folium Nelumbinis*)			
	3. Fresh Ce bai ye (*cacumen Platycladi*)			
	4. Fresh Zao jiao (*fructus Gleditsiae sinensis*)			

5	Jia Gu San: 1. Gu Sui Bu (*Rhizoma drynariae*)	Crush the ingredients into powder and mix with Vaseline to create an ointment. Apply the ointment to the painful area - Use topically	Promoting bone healing	[51]
	2. Xue jie (*sanguis Draconis*)			
	3. Borax			
	4. Dang gui (*radix Angelicae sinensis*)			
	5. Ru xiang (*gummi Olibanum*)			
	6. Mo yao (*Myrrha*)			
	7. Xu duan (*radix Dipsaci*)			
	8. Zi ran tong (*Cuprum*)			
	9. Da huang (*radix et rhizoma Rhei*)			
	10. *Blatta orientalis*			

**Table 4:** Examples of traditional Vietnamese medicine (TVM) remedies intended for the external (topical) or oral treatment of bone fractures. The present examples are classified as other documented TVM and Asian remedies.

No	Remedy	Preparation method/administration	Indication	Ref.
1	Gu She Wei Sa fang: 1. Ma qian zi (S*emen Strychni*) (12 g)	Grind the ingredients into a fine powder, mix with vinegar or alcohol, fry the mixture until warm then apply to the injured area. Change the bandage every 24 h - Use topically	Promote blood circulation and regeneration of bone tissue	[50]
	2. Sheng Cao Wu (*Radix Aconiti Preparata*) (8 g)			
	3. Long nao (*Borneolum*) (4 g)			
	4. Rou Gui (*cortex Cinnamomi*) (8 g)			
	5. Bing pian (*Borneolum*) (4 g)			
	6. Ding xiang *(flos Caryophylli*) (4 g)			
	7. Chi shao (*Radix Paeoniae Rubra*) (4 g)			
	8. Mo yao (*Myrrha*) (8 g)			
	9. Ru xiang (*gummi Olibanum*) (8 g)			
	10. Xi xin (*Asarum sieboldii*) (8 g)			
	11. Da huang (r*adix et rhizoma Rhei*) (8 g)			
	12. Sheng Chuan Wu (*Radix Aconiti Preparata*) (8 g)			
	13. Tian nan xing (*rhizoma Arisaematis*) (8 g)			
	14. Gan cao (*radix et rhizoma Glycyrrhizae*) (8 g)			

2	1. Roots of *Morinda citrifolia* (16 g)	Decoct with 3 bowls of water (750–800 mL) until it reduces to 150–250 mL, mix with a bit of alcohol, then consume - Use orally	Bone fractures	[50]
	2. *Zingiber zerumbet* (8 g)			
	3. Scoparia dulcis (12 g)			
	4. Can ger zi (*fructus Xanthii*) (12 g)			
	5. Bai mao gen (*rhizoma Imperatae*) (12 g)			
	6. *Cassia occidentalis* (12 g)			
	7. Aiye (*folium Artemisiae argyi*) (4 g)			
	8. Roots of *Cassia grandis* (12 g)			
	9. *Zingiber montanum* (8 g)			
	10. Xiang mao (*Cymbopogon citratus*) (12 g)			
	11. Niu jin cao (*herba Eleusine indicae*) (8 g)			
	12. Han lian cao (*herba Eclipta*) (12 g)			
	13. Ji xue cao (*Centella asiatica*) (12 g)			
	14. Sheng jiang (*Zingiber officinale*) (3 slices)			

3	1. Zhi jia hua (*Lawsonia inermis*) (10 g)	Decoct with 2 bowls of water (500 mL) until it reduces to 100 mL, consume the warm decoction before meals - Use orally	Bone fractures	[50]
	2. Xue jie (*Sanguis Draconis*) (12 g)			
	3. Su mu (*Lingum Sappan*) (10 g)			
	4. Aiye (*Folium Artemisiae argyi*) (12 g)			
	5. Jianghuang (*rhizoma Curcumae longae*) (10 g)			

4	1. Roots of *Morinda citrifolia* (32 g)	Crush and soak in 2 L of alcohol, ready for use after 7 days. Consume a small cup daily - Use orally	Bone fractures	[50]
	2. *Zingiber zerumbet* (16 g)			
	3. Chen pi ( *pericarpium Citri Reticulatae*) (12 g)			
	4. Roots of *Cassia grandis* (32 g)			
	5. *Zingiber montanum* (16 g)			
	6. Rougui (*cortex Cinnamomi*) (12 g)			

5	*Sterculia foetida*	The rough outer bark is scraped off, cleaned, finely sliced, and dried for making a decoction, or it can be used fresh and mashed for external applications. Dosage: 10–30 g per day - Use orally or topically	Bone fractures, toothaches, mumps, and dysentery	[51]

**Table 5:** Terpenoids and phenolic substances with potential uses in bone healing (adapted from [85, 93, 94]).

Class	Compound	Source	Proven effect	*Type of study*
Terpenoids	5-Hydroxy auraptene	*Esenbeckia grandiflora, Zanthoxylum rhoifolium*	Enhanced OB differentiation and activities	*In vitro*
	Abietic acid	*Pinus densiflora, Ceroplastes pseudoceriferus*	Reduced OC differentiation and activities and reduced LPS-dependent bone loss	*In vitro* and *in vivo*
	Alisol A, B, and C	*Alisma plantago aquatica*	Reduced OVX-related bone loss	*In vivo*
	Andrographolide	*Andrographis paniculata, Cymbopogon schoenanthus, Ginkgo biloba*	Induced OB differentiation and reduced OVX-related bone loss	*In vitro* and *in vivo*
	Bakuchiol	*Spiraea formosana, Cullen corylifolium,* etc.	Reduced OC differentiation and activities	*In vitro*
	Cafestol	*Coffea congensis, Diplospora dubia,* etc.	Reduced OC differentiation and activities and induced OB differentiation	*In vitro*
	Carnosic acid	*Salvia officinalis, Cuminum cyminum,* etc.	Reduced OC differentiation and activities and reduced OVX-related bone loss	*In vitro* and *in vivo*
	Catapol	*Veronica kellereri*, *Scutellaria racemosa*, etc.	Induced OB differentiation, increased bone regeneration, and decreased OVX-related bone loss	*In vitro* and *in vivo*
	Crocin	*Gardenia jasminoides, Crocus sativus,* etc.	Reduced metabolic syndrome-induced bone loss	*In vivo*
	Cycloastragenol	*Astragalus microcephalus, Astragalus coluteocarpus,* etc.	Increased OB differentiation and reduced natural and induced aged-related bone loss	*In vitro* and *in vivo*
	Decursin	*Angelica gigas, Phlojodicarpus villosus,* etc.	Reduced OC differentiation and activities and reduced LPS-induced osteolysis	*In vitro*
	Dehydrocostus lactone	*Ainsliaea uniflora, Costus,* etc.	Reduced OC differentiation and activities	*In vitro*
	Diosgenin	*Allium cernuum, Dioscorea hispida,* etc.	Reduced senescence-accelerated OXYS-related bone loss	*In vivo*
	Euphorbia factor L1	*Euphorbia lathyris*	Reduced OC differentiation and activities and reduced OVX-related bone loss	*In vitro* and *in vivo*
	Glycyrrhizic acid	*Hypomontagnella monticulosa, Glycyrrhiza pallidiflora,* etc.	Suppressed OC differentiation and activities and reduced OVX-related bone loss	*In vitro* and *in vivo*
	Hederagenin	*Rosa laevigata, Dipsacus inermis,* etc.	Reduced OC differentiation and activities and reduced OVX-related bone loss	*In vitro* and *in vivo*
	Kahweol	*Coffea congensis, Coffea sessiliflora,* etc.	Reduced OC differentiation and activities and induced OB differentiation	*In vitro*
	Kirenol	*Sigesbeckia glabrescens, S. orientalis, S. pubescens*	Reduced OC differentiation	*In vitro* and *in vivo*
	Limonin	*Raulinoa echinata, Citrus tankan,* etc.	Increased OB differentiation and reduced OVX-related bone loss	*In vitro* and *in vivo*
	Lupeol	*Camellia sinensis, Acanthus ilicifolius,* etc.	Reduced OC differentiation and activities and reduced 1*α*,25-(OH)2d3-induced bone loss	*In vitro* and *in vivo*
	Lycopene	*Pyracantha angustifolia, Allomyces javanicus,* etc.	Induced OB differentiation and reduced OVX-related bone loss	*In vitro* and *in vivo*
	Monotropein	*Galium rivale*, *Pyrola japonica*, etc.	Induced OB differentiation and reduced bone loss in LPS-treated OVX mouse	*In vitro* and *in vivo*
	Nomilin	*Microula sikkimensis, Citrus reticulata,* etc.	Reduced RANKL-dependent OC differentiation and activities	*In vitro*
	Oleanolic acid	*Salvia miltiorrhiza, Sambucus chinensis,* etc.	Reduced OC differentiation and activities	*In vitro*
	Ophiopogonin D	*Liriope muscari, Ophiopogon jaburan, Ophiopogon japonicus*	Induced OB differentiation, reduced OC differentiation and activities, and reduced OVX-related bone loss	*In vitro* and *in vivo*
	Oridonin	*Isodon japonicus, Isodon macrocalyx,* etc.	Increased OB differentiation and reduced OC differentiation and activities	*In vitro*
	Osthole	*Seseli hartvigii, Angelica japonica,* etc.	Reduced RANKL-mediated OC differentiation, induced OB differentiation, and enhanced bone growth and strength during fracture repair	*In vitro* and *in vivo*
	Swertiamarin	*Schultesia lisianthoides*, *Gentiana thunbergii*, etc.	Induced OB differentiation and reduced OC differentiation and activities	*In vitro* and *in vivo*
	Tanshinone IIA	*Salvia miltiorrhiza, Salvia glutinosa,* etc.	Suppressed RANKL-induced OC differentiation and activities and reduced OVX-related bone loss	*In vitro* and *in vivo*
	Toonaciliatin K	*Toona ciliata*	Reduced LPS-dependent OC differentiation and activities, reduced inflammation in carrageenan-induced paw edema rats, and antiarthritic effects in adjuvant arthritis rats	*In vitro* and *in vivo*
	Tubeimoside I	*Bolbostemma paniculatum*	Reduced OC differentiation and activities and reduced diabetes Type II-induced bone loss	*In vitro* and *in vivo*
	Ursolic acid	*Salvia miltiorrhiza, Camellia sinensis,* etc.	Reduced OC differentiation and activities and reduced OVX-related bone loss	*In vitro* and *in vivo*
	β-Ecdysone	*Silene wallichiana, Helleborus torquatus,* etc.	Selective estrogen receptor modulator eliciting prostimulatory effects on the bone cells; increased OB proliferation	*In vitro* and *in vivo*

Phenolic substances	2,3,5,4′-tetra-hydroxy- stilbene-2-O-β-D-glycoside	*Radix Polygoni multiflori*	Reduced H2O2-dependent OB apoptosis and induced OB differentiation and activities.	*In vitro* and *in vivo*
	4-O-Methylhonokiol	*Magnolia officinalis*, *Illicium simonsii*, etc.	Reduced OC differentiation	*In vitro*
	Aloin	*Aloe ferox*, *Aloe africana*, etc.	Induced OB differentiation	*In vitro*
	Apigenin	*Camellia sinensis*, *Apis*, etc.	Inhibited OB and OC differentiation and reduced OVX-related bone loss	*In vitro* and *in vivo*
	Arctiin	*Saussurea macrota*, *Saussurea salicifolia*, etc.	Reduced OC differentiation	*In vitro* and *in vivo*
	Baicalein	*Lepisorus ussuriensis*, *Scutellaria prostrata*, etc.	Reduced OC differentiation; promotes in late- but not in early-stage bone healing	*In vivo*
	Cajanin-stilbene acid	*Cajanus cajan*	Reduced OC differentiation and activities and reduced OVX-related bone loss.	*In vitro*
	Calycosin	*Bowdichia virgilioides*, *Glycyrrhiza pallidiflora*, etc.	Induced OB differentiation and activity	*In vitro*
	Cladrin	*Cladrastis platycarpa*, *Cladrastis sikokiana*, etc.	Reduced adipogenesis, inhibited OC activity, and reduced HFD-induced bone loss	*In vitro and in vivo*
	Corylin	*Ficus mucuso*, *Erythrina addisoniae*, etc.	Induced OB differentiation	*In vitro*
	Cudratrixanthone U	*Maclura tricuspidate*	Reduced OC differentiation and activities	*In vitro*
	Curcumin	*Curcuma longa*, *Curcuma xanthorrhiz*, etc.	Enhanced OB differentiation and activities, reduced glucocorticoid-related bone loss, increased pre-OC proliferation, decreased OC differentiation, and reduced OVX-related bone loss	*In vitro* and *in vivo*
	Cyanidin	*Camellia sinensis*, *Viburnum rafinesquianum*, etc.	Reduced adipocyte differentiation and induced chondrocyte differentiation	*In vitro*
	Daidzein	*Streptomyces padanus*, *Glycine soja*, etc.	Induced OB differentiation and inhibited OC differentiation	*In vitro*
	Delphinidin	*Camellia sinensis*, *Viburnum rafinesquianum*, etc.	Reduced adipocyte differentiation and induced chondrocyte differentiation	*In vitro*
	Denbinobin	*Dendrobium moniliforme*, *Dendrobium plicatile*, etc.	Inhibited NF-*κ*B signaling and reduced inflammatory mediator production	*In vitro*
	Desoxyrhapontigenin	*Alpinia hainanensis*, *Dracaena cochinchinensis*, etc.	Reduced OC differentiation and activities, reduced oxidative stress, and reduced LPS-induced osteolysis	*In vitro* and *in vivo*
	Echinacoside	*Jasminum mesnyi*, *Echinacea angustifolia*, etc.	Enhanced OB differentiation and activities and reduced diabetic-related bone loss	*In vitro* and *in vivo*
	Ellagic acid	Widely distributed in plants	Reduced OC differentiation and activities and reduced OVX-related bone loss	*In vitro* and *in vivo*
	Emodin	Various Chinese herbs	Induced OB differentiation, reduced cadmium-induced osteolysis, and reduced IBD-dependent bone loss	*In vitro* and *in vivo*
	Equol	*Punica granatum*	Induced OB proliferation and differentiation	*In vitro*
	Formononetin	*Dalbergia nigrescens*, *Glycyrrhiza pallidiflora*, etc.	Reduced HFD-induced bone loss, induced OB activity, and increased bone regeneration of femoral drill-hole injury in OVX mouse	*In vitro* and *in vivo*
	Gallic acid	Widely distributed in plants	Increased bone regeneration	*In vivo*
	Gambogic acid	*Garcinia hanburyi*	Reduced OC differentiation and activities and reduced OVX-related bone loss.	*In vitro and in vivo*
	Genistein	*Salvia hispanica*, *Glycine soja*, etc.	Induced OB differentiation and reduced ORX-related bone loss	*In vitro and in vivo*
	Gomisin A	*Kadsura interior*, *Kadsura heteroclita*, and *Schisandra chinensis*	Induced OB differentiation	*In vitro*
	Hesperidin	*Humulus lupulus*, *Ficus erecta* var. *beecheyana*, etc.	Induced OB differentiation, reduced TNF-*α* and NF-*κ*B expression induced by Type I DM, and ameliorated bone structure	*In vitro* and *in vivo*
	Honokiol	*Magnolia officinalis*, *Illicium simonsii*, etc.	Induced OB differentiation and reduction of methylglyoxal-mediated oxidative stress	*In vitro*
	Icariin	*Epimedium brevicornu*, *Epimedium truncatum*, etc.	Induced OB differentiation; reduced OC differentiation, OVX-related bone loss, and RANKL-induced OC differentiation; induced OB differentiation; reduced adipocyte differentiation; and reduced LPS-related bone loss	*In vitro* and *in vivo*
	Luteolin	*Camellia sinensis*, *Codonopsis lanceolata*, etc.	Induced OB differentiation	*In vitro* and *in vivo*
	Magnolol	*Magnolia henryi*, *Magnolia officinalis*, etc.	Reduced RANKL expression of OBs, reduced OC differentiation, reduced OC differentiation, and reduced OVX-related bone loss	*In vitro* and *in vivo*
	Malvidin	*Camellia sinensis*, *Vicia faba*, etc.	Induced OB differentiation	*In vitro*
	Mangiferin	*Rigidella*, *Polygala tenuifolia*, etc.	Induced OB differentiation and reduced OC differentiation	*In vitro* and *in vivo*
	Methyl gallate	*Camellia sinensis*, *Paeonia emodi*, etc.	Reduced OC differentiation and activities	*In vitro* and *in vivo*
	Petunidin	*Vicia faba*, *Pereskia aculeata*, etc.	Induced OB differentiation, reduced OC differentiation and activity, and reduced sRANKL-induced bone loss	*In vitro* and *in vivo*
	Puerarin	*Bupleurum chinense*, *Pueraria calycina*, etc.	Reduced OVX-related bone loss	*In vitro*
	Quercetin	Widely distributed in plants	Induced OB differentiation and reduced the inhibition of TNF-*α*-dependent OB differentiation	*In vitro* and *in vivo*
	Resveratrol	Red grapes	Induced OB differentiation and activities, reduced OC differentiation and activities, increased OC apoptosis, reduced OVX-induced oxidative stress damage, and reduced OVX-related bone loss	*In vitro* and *in vivo*
	Rubiadin-1-methyl ether	*Hymenodictyon orixense, Coelospermum paniculatum,* etc.	Reduced OC differentiation	*In vitro*
	Rutin	Widely distributed in plants	Induced OB differentiation	*In vitro*
	Schisandrin A	*Schisandra bicolor*, *Schisandra sphenanthera*, etc.	Reduced OC differentiation and activities and reduced OVX-related bone loss	*In vitro* and *in vivo*
	Sesamin	*Otanthus maritimus*, *Apis*,etc.	Reduced OC differentiation	*In vitro*
	Syringic acid	*Paeonia obovata*, *Rhinacanthus nasutus*, etc.	Induced OB differentiation, reduced OC differentiation and activities, and reduced OVX-related bone loss	*In vitro* and *in vivo*
	Thymoquinone	*Origanum dictamnus,* Ranunculaceae, etc.	Reduced OC differentiation and activities, reduced oxidative stress, reduced LPS-induced osteolysis, and reduced OVX-related bone loss	*In vitro* and *in vivo*
	Vanillic acid	*Camellia sinensis*, *Paeonia obovata*, etc.	Reduced OC differentiation and activities and reduced OVX-related bone loss	*In vitro* and *in vivo*

## Data Availability

The data supporting this review are from previously reported studies and datasets, which have been cited.

## Appendix A. TCM and Bone Healing

The written prescriptions of Hua Tuo (translated by Tuong Quan) [36] for the treatment of conditions related to musculoskeletal injuries (see Tables A1).

## Appendix B. TVM and Bone Healing

In this, examples of TVM remedies intended for the external (topical) or oral treatment of bone fractures are presented. The present examples are (I) documented in an ancient medical literature, (II) collected from the experiences of TVM practitioners today, or (III) classified as other documented TVM and Asian remedies.

## B.1. Examples From Ancient TVM Literature

The remedies are described according to the notes of Tue Tinh in Miraculous Effects of Southern Medicine (Nam Dược Thần Hiệu) [46] and Le Huu Trac in Practice of the Lazy Master of Hai Thuong (Hải Thượng Lãn Ông Y tông Tâm lĩnh) [47] (see Table A2).

## B.2. Examples of Traditional Remedies Collected From TVM Practitioners Today

The rhizome of *Drynaria fortunei*, a species that belongs to the Polypodiaceae family, is commonly used in the treatment of bone fractures and bone pain. In fact, the dried rhizome of *Drynaria fortunei* was named Gu Sui Bu because of its bone-related therapeutic effect. It has a bitter taste and warm property, which is attributed to the heart and kidney meridians. According to ancient pharmacological texts, this herb can detoxify bone-related toxins, alleviate pain from blood stasis caused by “bad qi,” cure depletion of the five Zang organs (heart, liver, spleen, lung, and kidney) and extreme deficiency involving six essential components (vital essence, bones, blood, tendons, semen, and qi) and treat paralysis and numbness in limbs. It also addresses symptoms like heat above and cold below, tonifies kidneys, alleviates tinnitus, eases dental pain, treats long-term kidney deficiency, relieves bone and tendon pain, aids in bone fractures, promotes blood circulation and regulates blood flow [48, 113].  Table A3 lists some remedies that use Gu Sui Bu as one of the main ingredients.

## B.3. Examples of Other Documented TVM and Asian Remedies

There is a nonsurgical field in TVM applied within traditional martial arts, called “Y Võ” (medicine in martial arts). This specialty focuses on issues that arise during training, such as sprains, dislocations, fractures, soft tissue injuries, commonly known as “Trật đả” (Fall-Hit or Die da). Martial arts masters, concurrently serving as injury treatment specialists, accumulate extensive experience in managing injuries to assist their learners in recovering from training-related traumas. In addition to procedures such as muscle and bone manipulation, various herbal remedies are employed to facilitate the recovery process [50, 51]. The following examples describe TVM remedies intended for injuries derived from tripping, beating, falling from a height, or severe impact leading to fractures, sprains, and dislocations.

From some of the TVM remedies mentioned above, it can be seen that traditional Asian medicine in general, and Vietnamese medicine in particular, holds a rich treasure trove of experience in treating musculoskeletal injuries. This serves as a valuable source of inspiration for future research aimed at elucidating its effectiveness in the bone healing process (see Table A4).

## Appendix C: Terpenoids and Phenolic Substances With Potential Uses in Bone Healing (see Table A5)

Terpenoids and phenolic substances with potential uses in bone healing [85, 93, 94] (see Table A5).
